# Comparison of Decellularization Protocols for Preparing a Decellularized Porcine Annulus Fibrosus Scaffold

**DOI:** 10.1371/journal.pone.0086723

**Published:** 2014-01-24

**Authors:** Haiwei Xu, Baoshan Xu, Qiang Yang, Xiulan Li, Xinlong Ma, Qun Xia, Yang Zhang, Chunqiu Zhang, Yaohong Wu, Yuanyuan Zhang

**Affiliations:** 1 Department of Spine Surgery, Tianjin Hospital, Tianjin, China; 2 Graduate School, Tianjin Medical University, Tianjin, China; 3 Cell Engineering Laboratory of Orthopaedic Institute, Tianjin Hospital, Tianjin, China; 4 School of Mechanical Engineering, Tianjin University of Technology, Tianjin, China; 5 Wake Forest Institute for Regenerative Medicine, Wake Forest University School of Medicine, Winston-Salem, North Carolina, United States of America; National University of Ireland, Galway, Ireland

## Abstract

Tissue-specific extracellular matrix plays an important role in promoting tissue regeneration and repair. We hypothesized that decellularized annular fibrosus matrix may be an appropriate scaffold for annular fibrosus tissue engineering. We aimed to determine the optimal decellularization method suitable for annular fibrosus. Annular fibrosus tissue was treated with 3 different protocols with Triton X-100, sodium dodecyl sulfate (SDS) and trypsin. After the decellularization process, we examined cell removal and preservation of the matrix components, microstructure and mechanical function with the treatments to determine which method is more efficient. All 3 protocols achieved decellularization; however, SDS or trypsin disturbed the structure of the annular fibrosus. All protocols maintained collagen content, but glycosaminoglycan content was lost to different degrees, with the highest content with TritonX-100 treatment. Furthermore, SDS decreased the tensile mechanical property of annular fibrosus as compared with the other 2 protocols. MTT assay revealed that the decellularized annular fibrosus was not cytotoxic. Annular fibrosus cells seeded into the scaffold showed good viability. The Triton X-100–treated annular fibrosus retained major extracellular matrix components after thorough cell removal and preserved the concentric lamellar structure and tensile mechanical properties. As well, it possessed favorable biocompatibility, so it may be a suitable candidate as a scaffold for annular fibrosus tissue engineering.

## Introduction

Disc degenerative disease is generally thought to be the main cause of chronic low back pain, which has a lifetime prevalence of 80% in the general population and causes a huge public health burden in industrialized countries [1]. Current treatments ranging from conservative management to invasive procedures are primarily palliative and seek to eliminate the pain generated by ruptured or herniated disks but do not attempt to restore disc structure and function [2]. Tissue-engineering techniques have emerged as a promising therapeutic approach to treat degenerative discs by replacing the damaged tissue with a biomaterial and appropriate cells [3].

The scaffold is a major component in tissue engineering. Cells live and proliferate in the scaffold, which can perform a variety of functions lacking in damaged tissue *in vivo*. An ideal scaffold is necessary in annulus fibrosus (AF) tissue engineering. It should have good biocompatibility, moderate porosity and proper degradation rate and be similar to natural AF in composition, shape, structure and mechanical properties [4].

The AF is a multi-lamellar fibrocartilagenous ring, comprised primarily of collagen and proteoglycans. It consists of 15–25 concentric layers within which the collagen fibers lie parallel to each other at approximately a 30° angle to the transverse plane of the disc but in alternate directions in successive layers [5]. The widths of lamellae in AF differ from outer to inner layers, being thicker in the inner than the outer layers. Meanwhile, the numbers of lamellae vary circumferentially, with the greatest number in the lateral region of the disc and the smallest in the posterior region [6]. The AF contains mainly types I and II collagen. The outer AF contains mostly type I and the inner AF contains mainly type II, for a decrease in ratio of types I to II collagen from the outer to inner AF [7]. However, water and proteoglycan content increase from the outer to inner AF [8].

The structure of AF is complicated and the components are distributed unevenly, so fabricating an artificial scaffold identical to AF in components and structure is difficult. To date, none of the scaffold designs used for AF tissue engineering, including polyamide nanofibers, alginate/chitosan hybrid fiber, demineralized bone matrix gelatin/polycaprolactone triol malate, and demineralized and decellular bone, have been able to replicate the composition and lamellar structure of AF. An ideal AF scaffold is the goal.

With the development of decellularization technology, tissue-specific extracellular matrix (ECM) as a complete novel biomaterial has attracted the attention of many researchers. ECM scaffolds and substrates are ideal candidates for tissue engineering because in our body, cells are surrounded by ECM. The ECM functions as a support material and also regulates cellular functions such as cell survival, proliferation, morphogenesis and differentiation. Moreover, the ECM can modulate signal transduction activated by various bioactive molecules such as growth factors and cytokines. Ideally, scaffolds and substrates used for tissue engineering and cell culture should provide the same or similar microenvironment for seeded cells as existing ECM *in vivo*. Decellularized matrices have been widely used for engineering functional tissues and organs such as cartilage, skin, bone, bladder, blood vessels, heart, liver, and lung [9]–[14] and have achieved impressive results.

Because acellular matrixes have been used for tissue engineering and clinical purposes, we wondered whether acellular AF could preserve the ECM, microstructure and biomechanical properties of native AF as ideal scaffold material for tissue-engineered AF. We found no evidence of decellularized AF in the literature, so we investigated a decellularization method suitable for AF. We compared 3 decellularization methods that are widely used and are effective in tissue or organ decellularization. We aimed to determine which method was advantageous in cell removal and preserving the ECM components, structure and mechanical properties of natural AF for an ideal scaffold for AF tissue engineering.

## Materials and Methods

### AF Preparation

We obtained animal material from the Animal Experimental Room of Tianjin Hospital. All animal experiments were approved by the Animal Experimental Ethics Committee of Tianjin Hospital and the animals were treated according to the experimental protocols under its regulations. Fresh pig tails were transported to the laboratory within 2 h after slaughter. AF were dissected from the intervertebral discs in pig tails. All surrounding tissues were carefully removed by use of scissors, and then AF samples were washed in phosphate-buffered saline (PBS) to remove excess blood. Specimens (external diameter 9∼11 mm, thickness 4.5∼5.5 mm) were randomly divided into 4 groups and treated as follows.

### Decellularization Methods

Triton X-100. Pig AF was placed in hypotonic Tris-HCl buffer (10 mM, pH 8.0) with 0.1% ethylenediamine tetraacetic acid (EDTA; Sigma) and 10 KIU/ml aprotinin (Sigma) at 4°C for 48 h. Then AF samples were agitated in Tris-HCl buffer with 3% Triton X-100 (Sigma), 0.1% EDTA and 10 KIU/ml aprotinin at 4°C for 72 h. The solution was changed every 24 h. Then AF samples were incubated with 0.2 µg/mL ribonuclease A (RNase A; Sigma) and 0.2 mg/mL desoxyribonclease I (DNase I; Sigma) at 37°C for 24 h. Finally, decellularized AF was washed with PBS for 24 h to remove residual reagents. All steps were conducted under continuous shaking [11], [15]–[17].

SDS. Pig AF was frozen at −80°C for 3 h and thawed at room temperature for 4 h. After 3 cycles of freezing-dissolving, AF samples were decellularized with 10 mM Tris-HCl buffer containing 0.5% SDS (Sigma), 0.1% EDTA and 10 KIU/ml aprotinin at room temperature for 72 h. The decellularization solution was refreshed every 24 h. Decellularized AF was incubated with 0.2 µg/mL RNase A and 0.2 mg/mL DNase I at 37°C for 24 h, then washed with PBS for 24 h to remove residual reagents. All steps were conducted under continuous shaking [12], [14], [18].

Trypsin. Pig AF were incubated under continuous shaking in trypsin/EDTA (0.5% trypsin and 0.2% EDTA; both Sigma) in hypotonic Tris-HCl buffer, together with RNase A (20 µg/ml) and DNase I (0.2 mg/ml) at 37°C for 72 h. The trypsin/EDTA solution was changed every 24 h. Then decellularized AF was washed with PBS for 24 h under shaking for removal of residual substances [19]–[21].

Control Group. Fresh pig AF was stored at −20°C.

### Histology

After decellularization, tissue specimens (n = 10) were fixed in 10% (v/v) neutral buffered formalin, dehydrated with a graded ethanol and embedded in paraffin wax, cut into sections of 5.0 µm by use of a microtome and mounted on glass slides. Haematoxylin and eosin (H&E) staining was used to evaluate the cellular content and general structure of the AF. Nucleic acids were stained with Hoechst 33258 dye (Sigma). Proteoglycan was visualized by Toluidine blue staining and Safranin O staining. Sirius red stain was used to visualize collagen distribution and orientation.

### Immunofluorescence Examination

Specimens for immunofluorescence stain were mounted with OCT compound and cryosectioned at 10 µm thick. After rehydration by immersion in PBS for 10 min, sections were incubated with a monoclonal antibody against collagen I (Shiankexing, Beijing) at 4°C overnight, followed by extensive washes with PBS, then incubated with FITC-conjugated IgG antibody (Sigma) for 1 h at room temperature. After 3 washes in PBS, sections were observed by fluorescence microscopy.

### Scanning Electron Microscopy (SEM)

Decellularized or control AF samples were freeze-dried, cut along the transverse plane by use of a sharp blade, then loaded onto aluminum studs, coated with gold and examined under a field emission scanning electron microscope (1530VP, LEO, Germany). Morphological changes were compared before and after treatment.

### Rehydration Analysis

Water imbibition was quantified to compare potential changes in imbibition properties of decellularized and natural AF. Fresh and decellularized AF (n = 15) was immersed in PBS containing 10 KIU/ml aprotinin at 4°C for 24 h to achieve fully swollen and hydrated states. Samples were then freeze-dried, and the weight before and after freeze-drying was measured. The swelling ratio (%) of samples was calculated as (Ws-Wd)/Wd, where Ws is the sample weight after immersion in PBS and Wd is the sample weight after freeze-drying [13].

### Collagen Content

Collagen content was measured as described [22]. Samples (n = 10) were first lyophilized to a constant weight, then samples (30 mg dry weight) were acid-hydrolyzed with hydrochloric acid (HCl) at 100°C for 20 min and neutralized with sodium hydroxide (NaOH). Oxidation of standard and test solution was achieved by adding N-chloro-p-toluenesulfonamide sodium salt (Chloramine T; Sigma) followed by p-dimethylamino-benzaldehyde (Sigma), and the absorbance was read at 570 nm. The amount of hydroxyproline present in the test samples was determined against a standard curve.

### Glycosaminoglycan (GAG) Content

GAG content was quantified by the DMMB assay as described [23]. Briefly, samples (n = 10) were freeze-dried to a constant weight, and samples (10 mg) were digested in papain buffer (125 mg/ml papain, 5 mM cysteine–HCl, 5 mM disodium EDTA in PBS) at 60°C for 24 h. Then, 50 µl of each sample was mixed with 250 µl 1, 9-dimethyl-methylene blue (Sigma) in a 96-well microtiter plate and the absorbance was measured at 530 nm. The amount of GAG content was calculated by reference to a standard curve prepared using different concentrations of chondroitin sulfate sodium salt from shark cartilage (Sigma).

### Biomechanical Testing

**Figure 1:**
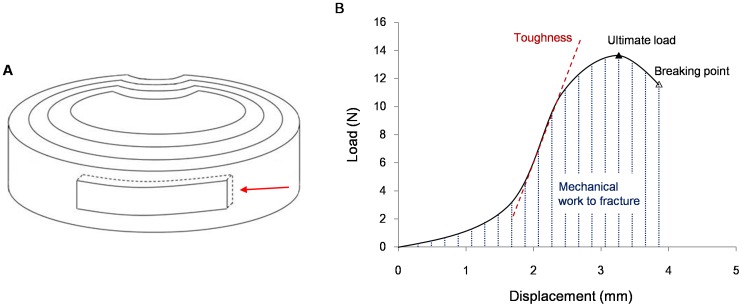
Schematic diagrams of specimens for tensile testing and load-displacement curve. (A) Schematic diagram of the intervertebral disc and locations of annulus fibrosus (AF) specimens for tensile testing. AF samples (arrow) were dissected from the outer zones of anterior regions, with the longest dimension in the circumferential direction. (B) Schematic diagram of load-displacement curve.

Mechanical test samples 15×4×1 mm were dissected from the outer anterior section of AF along circumferential direction (Fig. 1A). Before testing, samples were immersed in PBS (pH 7.4) for 4 h, then strips were mounted under zero strain onto frozen fixtures in a mechanical apparatus (Bose, Boston, USA) and the initial specimen length was recorded. The samples were then stretched to tensile failure at a rate of 1 mm/min. Samples were kept moist during testing by dropping normal saline solution on the specimens. All testing was conducted at room temperature.

For each specimen, ultimate load, stress, and strain; toughness; elastic modulus; and mechanical work to fracture were determined by computer and compared with the curve of load-displacement. A schematic diagram of the load-displacement curve is shown in Fig. 1B.

Ultimate load refers to the largest load value in the tensile process that can be read at the highest point of the load-displacement curve. It is a straightforward reflection of tissue strength but affected by the cross-sectional area of specimens. Under the same condition, ultimate load is positively related to the cross-sectional area. So, the ultimate load can be compared only in the same cross-sectional area.

Ultimate stress is a tensile parameter that excludes the influence of cross-sectional area. It refers to the amount of force per unit of initial cross-sectional area at tensile failure. Ultimate stress was calculated by dividing the maximum load by the original cross-sectional area of the specimen.

Ultimate strain was calculated by dividing the change in length by the initial length of the specimen.

Toughness is the slope of the ascending linear portion of the load-displacement curve. The greater the toughness, the harder the specimens is pulled off.

Elastic modulus refers to the stress needed to produce per unit of elastic deformation. It is one of the most commonly used indicators reflecting the tensile properties. Elastic modulus was calculated from the slope of the ascending linear region of the stress-strain curve.

Mechanical work to fracture is the work performed when the AF is stretched to fracture. Mechanical work to fracture was calculated by numerical integration of the area under the load-displacement curve in the left of breaking point.

### Cytotoxicity Assay

Depending on the above results, cytotoxicity study and subsequent experiments were conducted with samples of the Triton X-100 Group.

3(4,5-dimethylthiazole-2-yl)-2,5-diphenyltetrazolium-bromide (MTT; Sigma) assay was performed to determine the cytotoxicity of decellularized AF. Briefly, rabbit AF cells were seeded onto wells of flat-bottomed 96-well plates at 5×10^3^ cells/mL (200 µl per well). The plates were incubated for 24 h before the medium was replaced with control medium (positive control) and different concentrations (25%, 50%, 100%) of extracts prepared as described [24]. At days 1–6, the proliferation activity of the cells was determined by MTT assay. The optical density (OD) absorbance at 570 nm was determined with use of a microplate reader (RT-6000, Rayto, USA). Five replicates were considered per sample.

### Isolation and Culture of AF Cells

Lumbar spines were dissected aseptically from New Zealand white rabbits (female, 6 weeks old) killed under the guidelines specified by the Animal Experimental Ethics Committee of Tianjin Hospital. AF was separated from intervertebral discs with use of a blade, and all surrounding tissues (including muscles, tendons and nucleus pulposus) were carefully removed. The collected AF was cut into small pieces and digested with 0.25% collagenase (Sigma) for 6 h at 37°C. Cell suspensions were filtered through a nylon mesh and cultured in Dulbecco’s modified Eagle’s medium (DMEM; Gibco) containing 10% fetal bovine serum (FBS; Gibco) and 1% antibiotics at 37°C in a humidified atmosphere of 5% CO_2_. The medium was changed every 3 days. Cells at passage 2 were used in this study.

### Cell Seeding

Decellularized AF (Triton X-100 Group) was disinfected with 70% ethanol, thoroughly rinsed in sterile PBS for 24 h, and immersed in DMEM containing 10% FBS and 1% antibiotics for 24 h. The liquid on the surface of decellularized AF was dried by use of sterile filter paper, then 100 µl cell suspension containing 1×10^6^ AF cells was seeded into each decellularized AF by drop-wise addition onto the surface of the decellularized AF. At 1 h later, the decellularized AF was turned over and another 100 µl cell suspension was seeded onto the surface. The cell-containing constructs were incubated for 2 h before the culture medium was supplemented slowly for further culture. Culture medium was changed every 2 days.

### Cell Distribution and Viability Assessment

After 7 days of culture, the cell-seeded constructs were fixed in 10% (v/v) neutral buffered formalin, dehydrated with ethanol and embedded in paraffin wax. They were cut into sections of 5.0 µm by use of a microtome and stained with H&E to observe cell distribution in decellularized AF. The viability of cells seeded into scaffolds was detected by a live/dead assay kit (Invitrogen): live cells were stained with calcein AM (green) and dead cells with ethidium homodimer (EthD-1) (red). The constructs were incubated with live/dead dye at 37°C, 5% CO_2_, with saturated humidity for 30 min, then constructs were observed under a confocal microscope (TCS SP5 II, Leica, Germany) for cell viability.

### Statistical Analysis

Data analysis involved SPSS 16.0 (SPSS, Chicago, IL, USA). Results were expressed as mean ± SD. Differences between groups were assessed by one-way ANOVA, followed by Sceffe or Tamhane’s T2 tests for multiple comparisons. P<0.05 was considered statistically significant.

## Results

### Morphology and History

**Figure 2:**
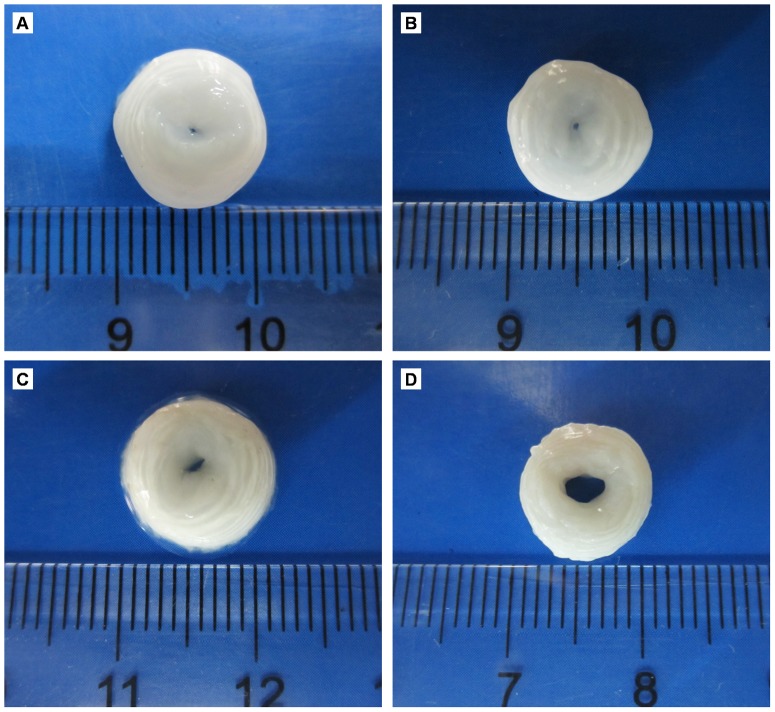
Representative macroscopic images of AF before and after decellularization. (A) Triton X-100, (B) SDS, (C) trypsin, (D) control.

Macroscopically, after decellularization, AF swelled and the central voids became smaller as compared with natural AF (Fig. 2A–D). The 3 decellularization groups did not differ macroscopically.

**Figure 3:**
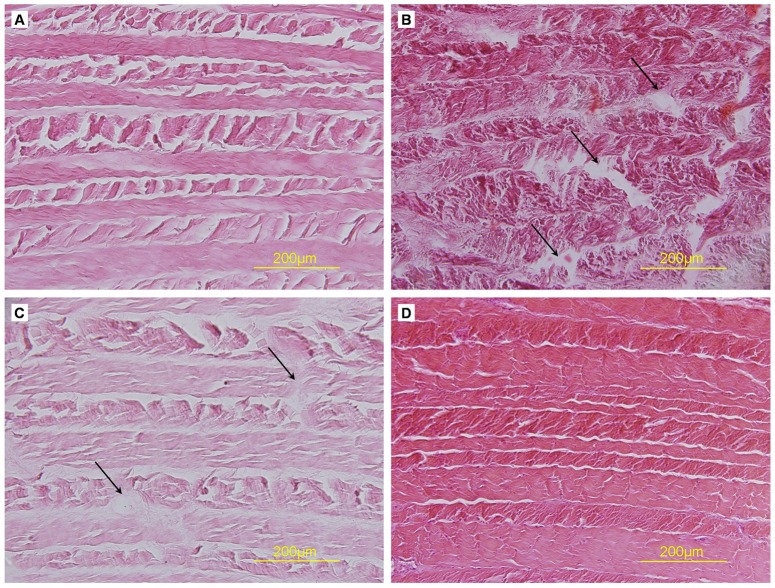
Hematoxylin and eosin (H&E) staining of cross-sections of AF samples. (A) Triton X-100, (B) SDS, (C) trypsin, (D) control. Collagen fiber fracture (arrows).

**Figure 4:**
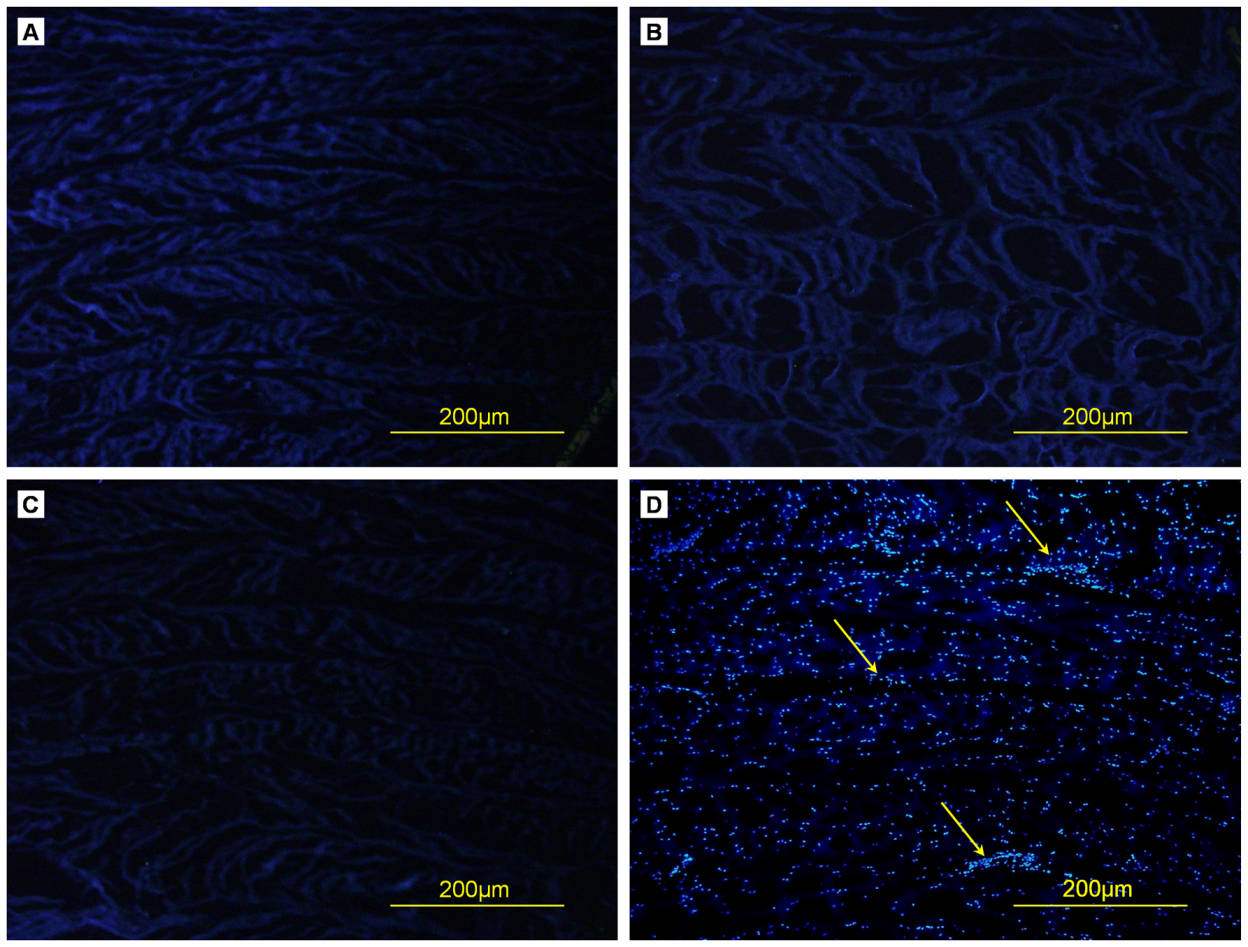
Hoechst 33258 staining of cross-sections of AF samples. (A) Triton X-100, (B) SDS, (C) trypsin, (D) control. DNA (arrows).

On H&E staining, control AF showed many cells scattered among collagen fibers, which were compact with an ordered arrangement (Fig. 3). Decellularized Triton X-100, SDS or trypsin samples showed no cells, and the mesh of collagen fibers was looser than in control samples. Triton X-100 and trypsin samples retained the concentric lamellar arrangements of collagen, similar to natural AF, but some fractured collagen fibers could be seen in trypsin samples. In SDS samples, lamellar arrangements of collagen were disturbed, with gaps between the collagen fibers. Results were similar with Hoechst 33258 staining (Fig. 4). Many blue fluorescent dots representing DNA were evenly distributed in natural AF, with none in Triton X-100, SDS or trypsin samples.

**Figure 5:**
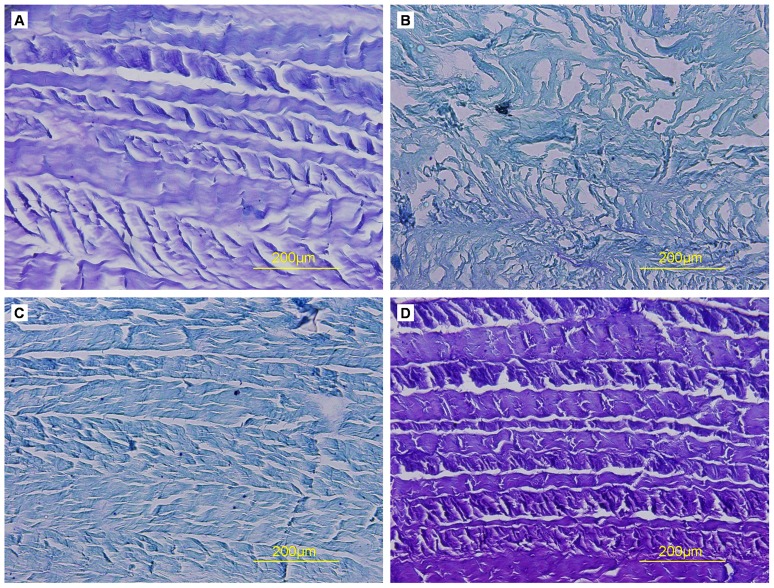
Toluidine blue staining of cross-sections of AF samples. (A) Triton X-100, (B) SDS, (C) trypsin, (D) control.

**Figure 6:**
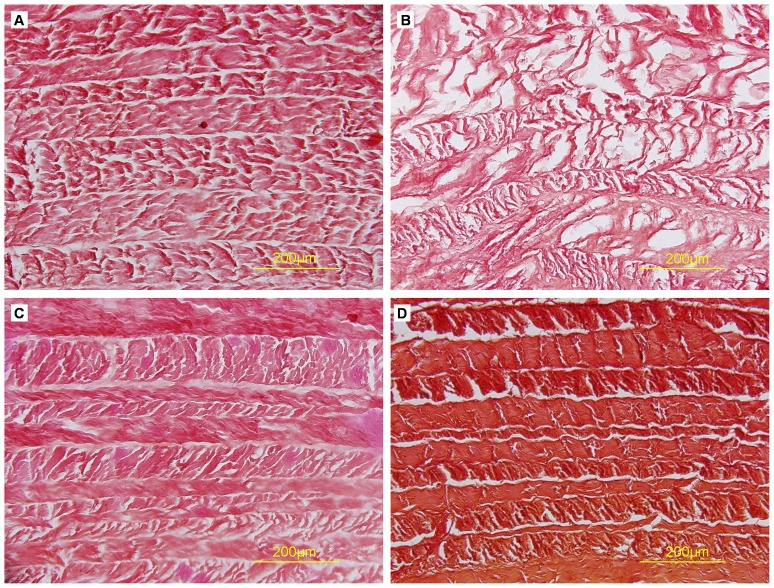
Safranin O staining of cross-sections of AF samples. (A) Triton X-100, (B) SDS, (C) trypsin, (D) control.

**Figure 7:**
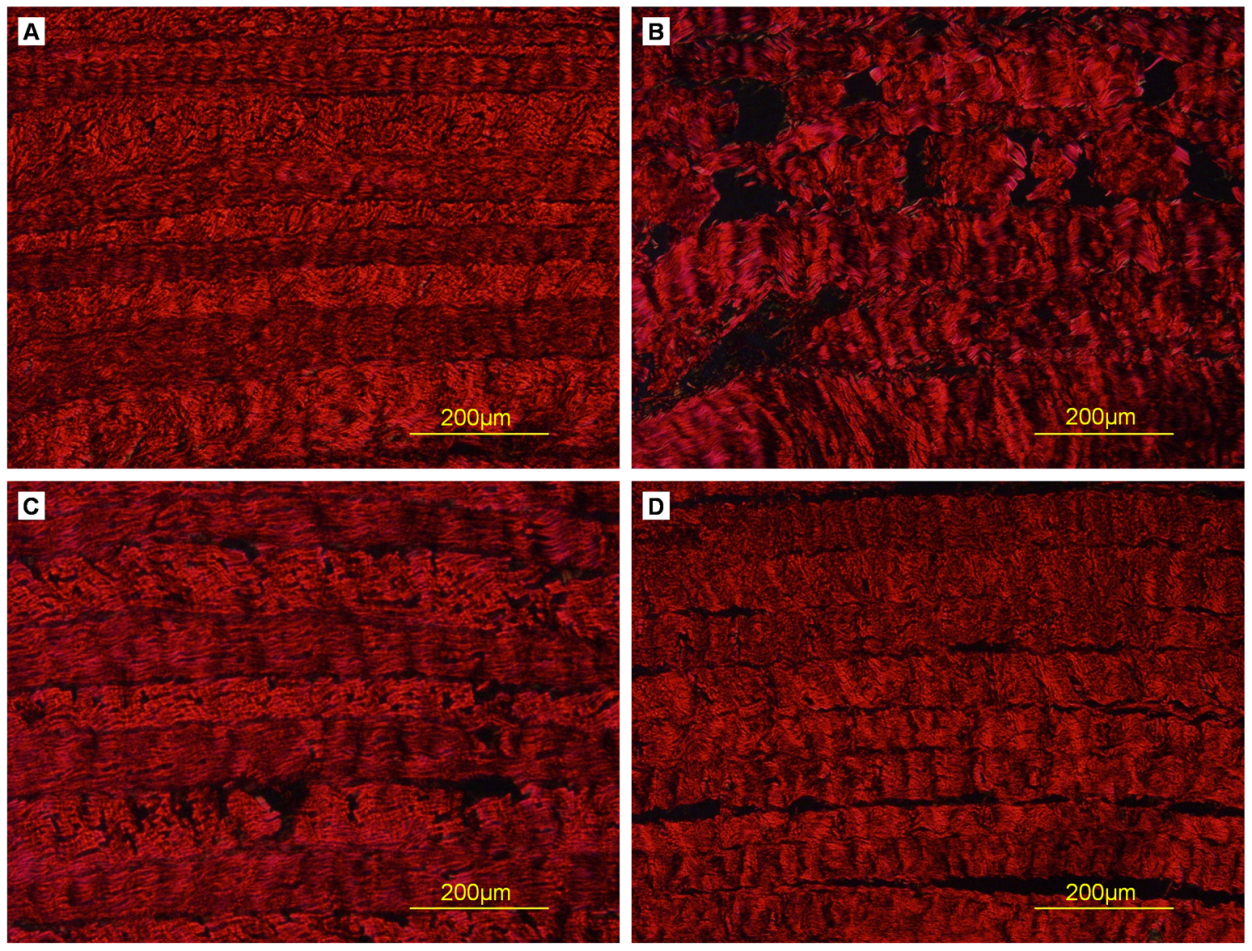
Sirius red stain of cross-sections of AF samples. (A) Triton X-100, (B) SDS, (C) trypsin, (D) control.

Toluidine blue and Safranin O staining showed that both natural AF and decellularized AF were rich in proteoglycans, but staining was less dense in decellularized than natural AF (Fig. 5,6). Proteoglycan content may have decreased during the decellularization process. Sirius red staining showed enriched collagen content in both natural and decellularized AF (Fig. 7).

### Immunohistochemistry

**Figure 8:**
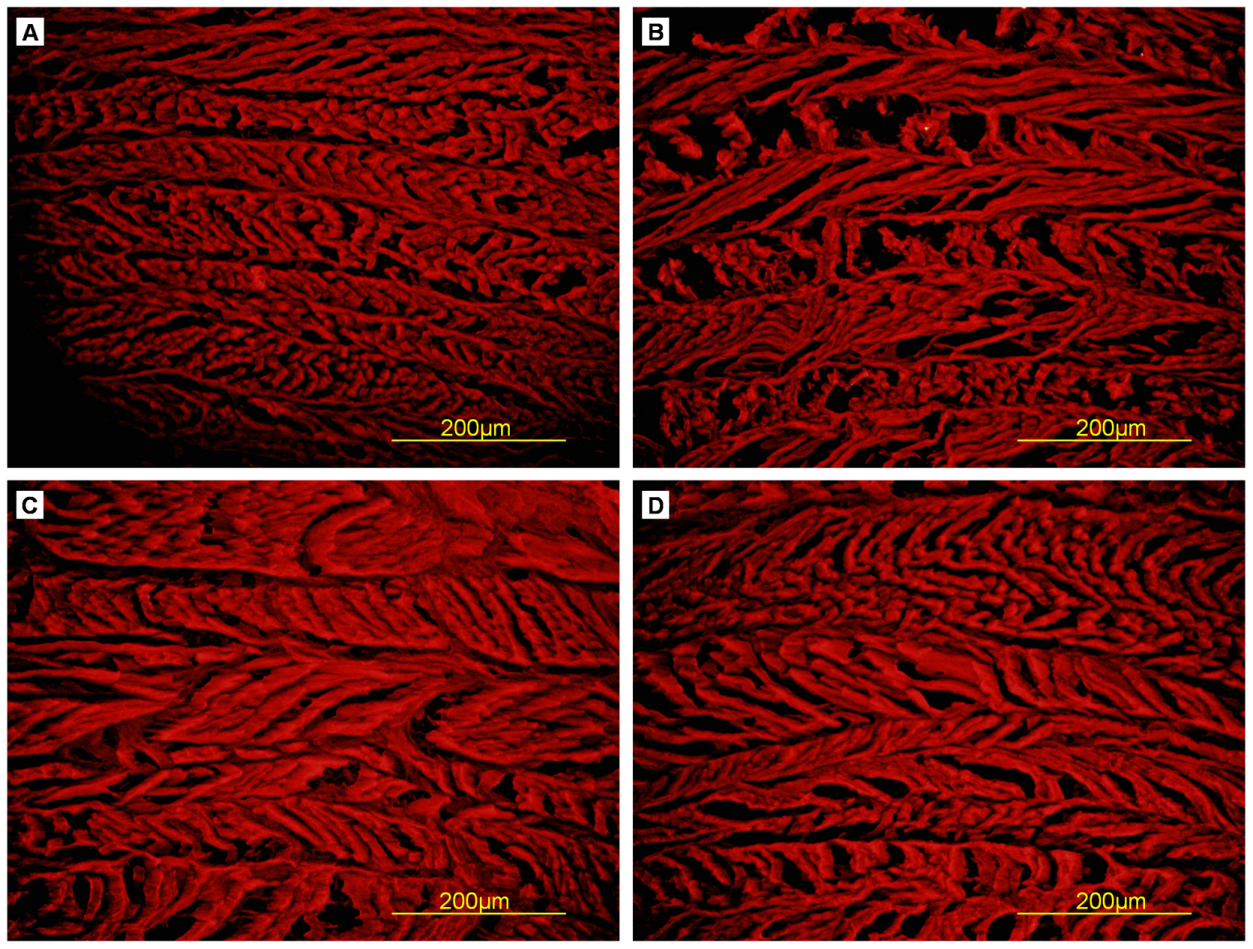
Collagen I immunoﬂuorescent staining of cross-sections of AF samples. (A) Triton X-100, (B) SDS, (C) trypsin, (D) control.

All samples were positive for collagen type I (Fig. 8), with no differences in staining density.

### SEM

**Figure 9:**
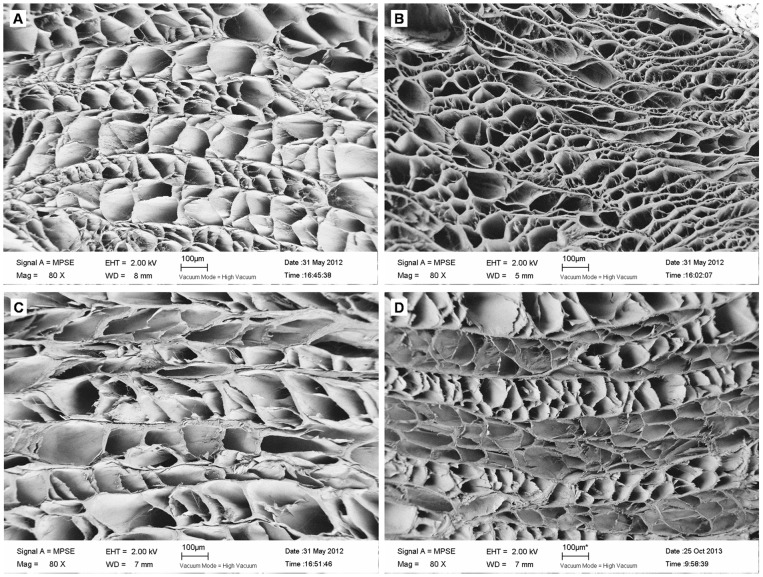
Scanning electron micrographs of cross-sections of AF samples. (A) Triton X-100, (B) SDS, (C) trypsin, (D) control.

In control samples, collagen fibers were arranged orderly, with a concentric lamellar structure (Fig. 9). Triton X-100 samples showed a concentric lamellar structure, with no difference from natural AF. However, the arrangement of collagen fibers was severely disturbed in SDS samples, with no lamellar structure. Trypsin samples retained the concentric lamellar structure, but the arrangement of collagen fibers was somewhat disorganized as compared with control and Triton X-100 samples.

### Hydration Results

**Figure 10:**
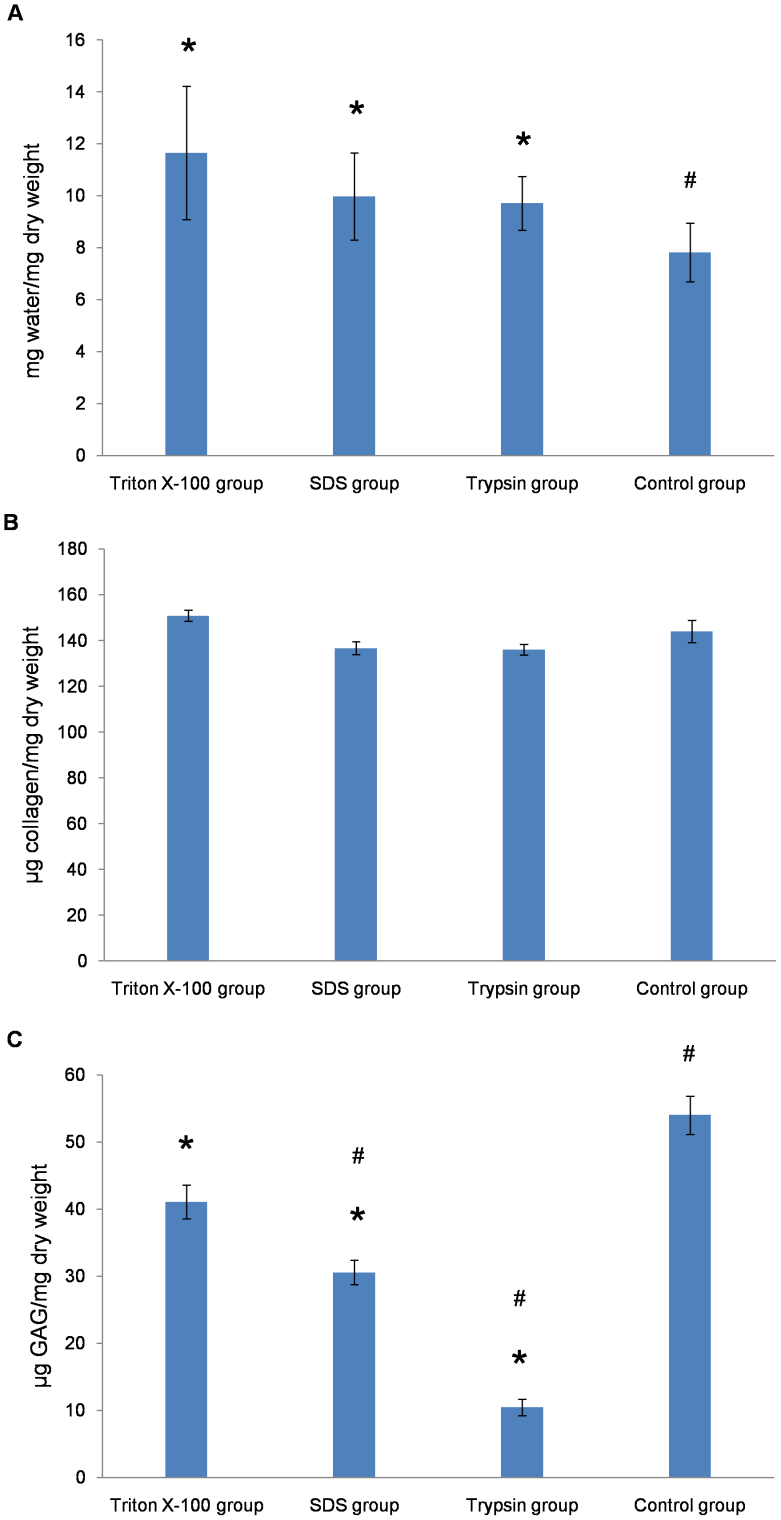
Water (A), collagen (B), and glycosaminoglycan (GAG) content (C) of AF. Data are mean ± SD. * =  p<0.05 compared to control, # = p<0.05 compared to Triton X-100.

The decellularized AF showed a high capacity to absorb water (Fig. 10A). The swelling ratios for decellularized AF in Triton X-100, SDS, and trypsin samples did not differ from each other (11.65±2.56, 9.97±1.68, 9.71±1.04 mg water/mg sample dry weight respectively), but swelling was greater than for control samples (7.81±1.13) (p<0.05), so decellularized AF contained significantly more water than natural AF. This water uptake was likely responsible for “pushing apart” areas of the collagen matrix throughout decellularized AF, leading to the appearance shown on H&E, Toluidine blue and Safranin O staining.

### Quantification of Collagen

The content of hydroxyproline was detected in samples for calculating collagen content. Control and decellularized AF samples did not differ in mean collagen content per mg of tissue (Fig. 10B).

### Quantification of GAG

GAG content was lower in decellularized than control AF samples (p<0.05; Fig. 10C). The GAG content in Triton X-100 samples was closest to that in natural AF, and higher than that in SDS or trypsin samples (p<0.05). GAG content was lower in SDS and trypsin than control samples.

### Biomechanical Testing

The ultimate load and stress values decreased as follows: Triton X-100> control>trypsin>SDS samples, with no significant difference between control and Triton X-100 or trypsin samples but a difference between control and SDS samples (P = 0.004, P = 0.012, Table 1). The ultimate strain values decreased as follows: Triton X-100> SDS>control>trypsin samples, with no significant difference among the 4 groups (P = 0.078). The toughness and elastic modulus values decreased as follows: trypsin>control>Triton X-100> SDS samples, with no significant difference between control and Triton X-100 or trypsin samples but a difference between control and SDS samples (P = 0.003, P = 0.008). The mechanical work to fracture values decreased as follows: trypsin>Triton X-100> control>SDS samples, with no difference between control and Triton X-100 or trypsin samples but a difference between control and SDS samples (P = 0.027).

**Table 1:** The biomechanical properties of annulus fibrosus with decellularization treatments.

Group	Ultimate load (N)	Ultimate stress (MPa)	Ultimatestrain (%)	Toughness(N/mm)	Elastic modulus(MPa)	Mechanical work to fracture (×10^−3^ J)
Triton X-100	24.52±3.83	6.02±0.83	0.41±0.05	15.58±1.62	28.89±5.50	30.85±5.15
SDS	11.27±2.68*	2.86±0.34*	0.39±0.07	5.45±1.10*	14.71±1.19*	16.23±4.27*
Trypsin	20.18±3.31	4.94±0.58	0.28±0.06	17.67±3.28	34.94±3.53	35.14±4.93
Control	22.98±2.10	5.86±1.13	0.34±0.05	17.00±2.89	30.71±5.47	29.62±5.26

* p<0.05, vs. control.

Data are mean±SD, n = 10 in each group.

### Cytotoxicity Assay

**Figure 11:**
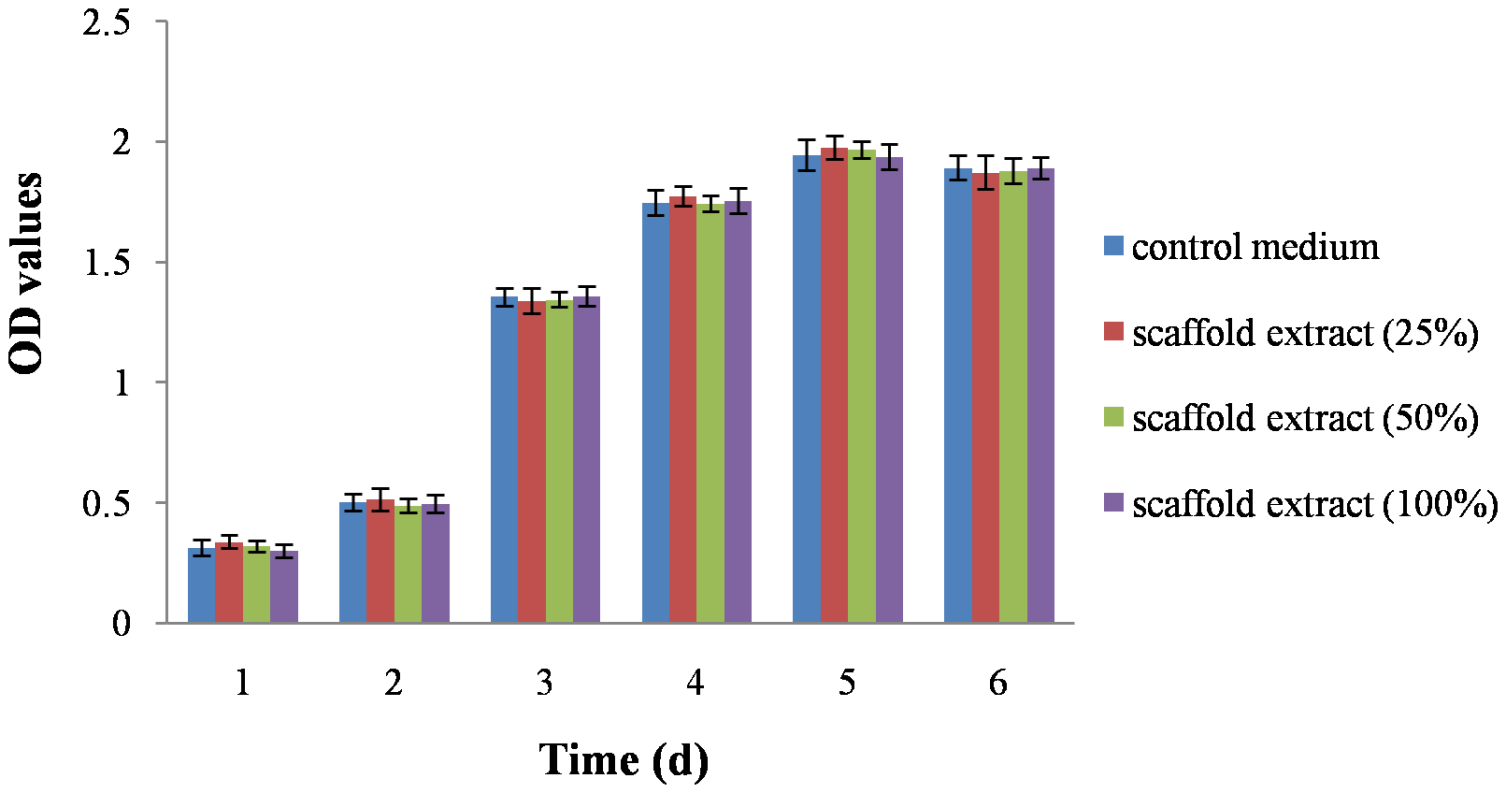
Cytotoxicity of decellularized AF. MTT assay of proliferation of AF cells cultured with different concentrations of scaffold extracts.

Different concentrations of extracts had no effect on cell proliferation, with no difference in OD values for the 4 groups at each time (P>0.05), so the decellularized AF were not cytotoxic (Fig. 11).

### Cell Distribution and Viability Assessment

**Figure 12:**
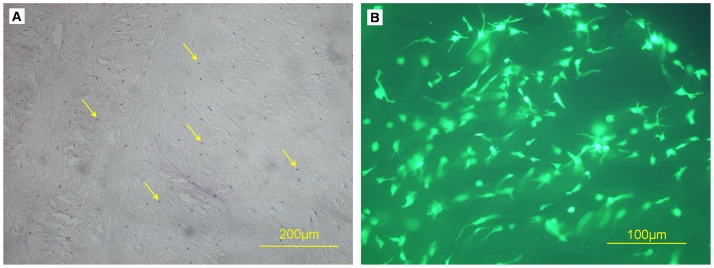
Recellularization of decellularized AF and Evaluation. (A) H&E staining of cell-containing constructs. AF cells (arrows). (B) Live/dead staining of cells seeded into decellularized AF. (Green: viable, red: necrotic).

After 7 days of culture, AF cells infiltrated the mid-horizontal plane of decellularized AF (Fig. 12A). Live/dead staining showed live cells evenly distributed in decellularized AF, with no dead cells (Fig. 12B).

## Discussion

In the present study, we explored the use of a non-ionic detergent (Triton X-100), an anionic detergent (SDS) and enzymatic agent (trypsin) to decellularize pig AF and compared the histological structure and biomechanical properties of decellularized AF as an ideal scaffold for AF tissue engineering. Triton X-100–treated AF retained the major ECM components after thorough cell removal, preserved the concentric lamellar structure and tensile mechanical properties, and possessed favorable biocompatibility, so it is a suitable candidate for producing scaffold material for AF tissue engineering.

The immunogenicity of acellular matrixes must be eliminated before they are used for tissue engineering. Cells are the main immunogenic factors in tissue. Histocompatibility antigens (human leukocyte antigen) are distributed on the surface of cell membranes in the form of lipoproteins or glycoproteins. They are genetically determined and differ among individuals within the same or different species. Histocompatibility antigens are recognized by T cells, and the tissue is attacked by the recipient host after transplantation of allogeneic cells. So, before ECM is used as scaffold, the cells must be removed to avoid immune rejection, inflammation, and potential transplant rejection [25]. Our H&E staining showed that all 3 decellularization agents removed cells. Furthermore, hochest 33258 staining, which emits blue fluorescence when bound to double-stranded DNA, showed no DNA in decellularized AF with the 3 agents. Therefore, use of the 3 agents was effective in AF decellularization. Previously, decellularization with Triton X-100 completely removed nuclear material in nerve, pericardium and bone [11], [16]–[17]; with SDS removed cells in meniscus, cornea and cartilage bone [12], [14], [18]; and with trypsin removed cells in dermal, aortic and aortic valve tissue [13], [15], [19], [21]. However, the cell removal efficacy of Triton X-100 is controversial: nuclear material was observed in tendon, artery, and ligament after decellularization with Triton X-100 solution [26]–[28]. The decellularization effect of Triton X-100 is related to the organization of the material. As well, concentrations of detergents affect decellularization efficiency. Recently Chan et al. [24] decellularized bovine intervertebral disc to create a natural intervertebral disc scaffold with 0.1% SDS. Many dead cells were left in the intervertebral disc on live/dead staining, whereas in our study, 0.5% SDS produced no cells in decellularized AF.

Collagen and GAG are the main components of the AF ECM. They play an important role in guiding cellular attachment, survival, migration, proliferation, differentiation [29]. The ideal decellularized AF ECM should contain collagen and GAG content close to that of natural AF. We calculated collagen content by presence of hydroxyproline in the test samples and found no difference between decellularized AF and control samples, which indicates no collagen lost in the decellularization process with Triton X-100, SDS or trypsin. However, GAG content was reduced with decellularization, especially with trypsin, and the GAG content was closest to that of the control with Triton X-100. The preservation of collagen and loss of GAG may be related to their relative position. Within and between the lamellae is a proteoglycan-rich ground substance [30]. The orderly arranged collagen fibers are embedded in a matrix rich in proteoglycan and GAG, which are exposed to decellularization solution and more likely to be lost during decellularization as compared with collagen [31]. Especially, trypsin has the ability of disconnecting the interactions between the matrix proteins, thus creating a more open matrix, which results in more GAG lost. Triton X-100 was superior to the other treatments in retaining collagen and GAG content.

AF is a multi-lamellar fibro-cartilagenous ring. The unique angle-ply architecture of AF is critical for withstanding multi-axial physiologic loads for normal function of the spine. After decellularization, H&E staining and SEM revealed a well-preserved concentric lamellae structure with Triton X-100. With trypsin, the concentric lamellar structure was slightly disturbed, with some collagen fractures seen on H&E staining. With SDS, the concentric lamellar structure was severely destroyed, with large gaps between collagen fibers, as seen on H&E staining and SEM. This finding was consistent with the reported features of SDS treatment. SDS, which has a negatively charged head-group and belongs to anionic detergents, can bind and denature both soluble and membrane-bound proteins. It can disrupt non-covalent bands within proteins and cause them to lose their native conformation. So SDS tends to disrupt the native tissue structure and causes decreased GAG concentration and loss of collagen integrity [25]. Cartmell et al. [32] decellularized rat tail tendons with Triton X-100, TnBP, and SDS. Treatment with SDS resulted in a pronounced opening of the spaces between the aligned collagen fibers regardless of concentration or treatment time. Kasimir et al. [33] treated aortic and pulmonary porcine valves with 0.1%, 0.03% and 0.01% SDS for 24 and 48 h. All concentrations completely removed cells. However, the matrix fibers were markedly disintegrated after 24 and 48 h. Reports about the effect of SDS differ. Liao et al. [15] processed porcine aortic valves with 0.1% SDS and preserved the trilayered structure of the native aortic valve. Therefore, the effects of SDS on tissue structure depend on the tissue substrate.

Mechanical property is an important parameter of the intervertebral disc. *In vivo*, intervertebral discs serve to support large spinal loads, which are combinations of tension, torsion, compression, and bending. The hydrostatic excess pressure in the nucleus pulposus caused by these loads generates large circumferential tensile stress in the surrounding AF [34]. The normal tensile mechanical properties of AF secure the nucleus pulposus in the right position and the intervertebral disc functions normally. AF exhibits regional variations in tensile mechanical properties [35]–[36]. The anterior AF has larger tensile values than the posterolateral annulus. Also, tensile values are larger in the outer than the inner regions of the annulus [8], [37]–[38]. These variations are generally attributed to inhomogeneity in tissue structure and biochemical composition. In the current study, the mechanical samples were all dissected from the outer anterior section of AF to eliminate the regional variation caused by inhomogeneous biochemical composition and structural organization.

We found no significant difference in ultimate load and stress, toughness, elastic modulus and mechanical work to fracture between Triton X-100, trypsin and control treatment; however, these parameters were lower with SDS than control treatment. The mechanical results have much to do with the structure of decellularized AF. Tensile properties are closely related to collagen content and arrangement [8]. The specimens treated with SDS had a seriously disturbed structure and broken collagen fibers, so their mechanical properties were lower than those of natural AF. The collagen content and arrangement of specimens was similar with Triton X-100 or trypsin and natural AF, for no difference between these 2 groups and natural AF.

We tested the biocompatibility of treated specimens, the most important feature of decellularized scaffolds for tissue engineering. In the decellularization process, a wide variety of chemicals are used, including EDTA, RNase A, and DNase I. If the chemicals remain within the tissue after decellularization, they will be toxic to host cells when the scaffold is implanted *in vivo*. So, we extensively washed specimens in PBS at the end of decellularization to clear any residual reagents and detected the toxicity of scaffolds by MTT and live/dead staining. MTT assay showed that scaffold extracts had no effect on cell proliferation, so the residual reagents were successfully removed. As well, live/dead staining showed that live cells were evenly distributed in the scaffold, with no dead cells, which also inferred that the scaffolds were non-cytotoxic.

Recently, Chan et al. [24] decellularized bovine intervertebral disc as a natural scaffold for intervertebral disc tissue engineering. In his study, a protocol for decellularizing bovine disc was investigated, in which SDS combining with freeze–thaw cycles has been applied, but lots of dead cells remained in the disc after decellularization. As we mentioned above, the decellularization effect of detergents is related to the organization of tissue. Intervertebral disc as a new tissue proposed for decellularized scaffold should be treated with different detergents to seek the optimal decellularization protocol. In 2011, the optimized decellularization procedure of NP tissue was studied by Mercuri JJ et al. [39]. To determine the optimal decellularization method suitable for AF, 3 protocols were applied in our study, including Triton X-100, SDS combined with freeze–thaw cycles and trypsin. The 3 protocols have been compared in cells removal, ECM content (collagen and GAG), microstructure (SEM) and tensile properties (ultimate load, stress, and strain; toughness; elastic modulus; and mechanical work to fracture). In our study, the concentric lamellar structure before and after decellularization was studied emphatically, for it is critical for withstanding multi-axial physiologic loads for normal function of the spine. We observed concentric lamellar structure of decellularized AF through history staining and SEM. While focus was concentrated on collagen fibril meshwork in Chan’s study. Besides, we recellularized AF cells into decellularized AF and observed cell proliferation and viability, which showed a high survival rate over 7 days, with cell penetration. While Chan et al. have focused on recellularization of decellularized NP with bovine NP cells.

## Conclusions

This study explored the possibility of using an AF matrix decellularlized with 3 agents as a tissue-engineered AF scaffold material. We compared decellularlized specimens with natural ones for cell removal efficiency, preservation of the matrix components, microstructure and mechanical function. Overall, Triton X-100–treated AF retained the major ECM components after thorough cell removal, preserved the concentric lamellar structure and tensile mechanical properties, and possessed favorable biocompatibility, so AF so treated would be a suitable candidate as a scaffold for AF tissue engineering. An *in vivo* study is still needed to determine whether the novel scaffold could have potential for intervertebral disc tissue engineering.
